# Muscle Activity during Handwriting on a Tablet: An Electromyographic Analysis of the Writing Process in Children and Adults

**DOI:** 10.3390/children10040748

**Published:** 2023-04-20

**Authors:** Sabrina Gerth, Julia Festman

**Affiliations:** Department of Research and Development in Teacher Education, University College of Teacher Education Tyrol, 6010 Innsbruck, Austria

**Keywords:** handwriting development, muscle activity, kinematic analysis of handwriting, school-age children

## Abstract

Handwriting is a complex task that includes planning the content and the execution of handwriting movements on paper or on a tool (e.g., a tablet). This execution depends on the involvement of specific muscles in the hand (distal) and arm (proximal). The present study combines the parallel recording of the writing process on tablets and the related muscle activity with electromyography to investigate the differences in handwriting movements in two groups. A total of 37 intermediate writers (third and fourth graders, mean age 9.6 years, SD 0.5) and 18 skilled adults (mean age 28.6 years, SD 5.5) participated in three handwriting tasks. The tablet data results replicate previous findings in handwriting research for the writing process. The muscle activity data reflected a differential relationship between distinct muscle activity and handwriting performance depending on the handwriting skill (intermediate or advanced writers). Furthermore, the combination of both methods revealed that skilled writers tend to involve rather distal muscles to control the pressure of the writing pen on the surface, whereas developing writers mainly use their proximal muscles to control the velocity of their handwriting movements. This research contributes to a deeper understanding of the underlying processes of handwriting and the development of efficient handwriting execution.

## 1. Introduction

Handwriting is a particularly important motor activity that takes children years to acquire. Writing by hand entails memorizing specific letter forms and sizes; with practice, handwriting movements become automatic [1,2,3]. Research on handwriting acquisition has shown that the development of fluent handwriting movements influences text generation skills and higher-level processes in writing (e.g., spelling, planning and revising) [4,5,6]. It has also been demonstrated that a proper education of graphomotor skills and handwriting could ease the transition from lower-level to higher-level writing processes. More generally, the development of fluid and legible handwriting is key to academic success [7].

Digitization has started to change our modes of writing (e.g., handwriting or typing) and has a direct impact on the education of handwriting [8]. The advance toward digitization in handwriting research is also visible in handwriting research methods. Whereas early studies focused on examining the handwriting product quality, there was a shift in recent years toward the assessment of handwriting process measures that are mainly recorded with tablets [9,10,11,12]. Tablets provide data on the real-time writing process as well as the writing product. Commonly extracted measures to assess the writing process are writing duration (i.e., the continuous time that the writing tool moves on the writing surface), writing velocity (i.e., the speed of the writing movement), writing pauses (i.e., the time when the writing tool is lifted from the surface), number of inversions in velocity (i.e., the number of accelerations and decelerations during writing, indicating automaticity of handwriting movements), and the writing pressure (i.e., the pressure of the writing tool on the surface). These measures are taken together as indicators of the level of handwriting skill and the fluency of handwriting movements.

Research on writing processes has demonstrated that beginning writers (e.g., children) take longer pauses in between writing bursts and use more strokes to complete the letters [13]. Writing experience can be observed in more smoothly executed handwriting movements (higher writing velocity) and in the ability to plan writing movements in advance with fewer pauses [14,15].

Previous studies also revealed that handwriting on a tablet is different from writing on paper, especially for beginning writers [11,12]. The lower friction of the tablet’s surface seemed to be a greater challenge for children than for adults, probably because their motor skills are not yet well trained.

Handwriting requires the manipulation of a writing tool (e.g., a pen) on a two-dimensional writing surface (e.g., paper). The tip of the pen must be held still or in motion above the surface to produce specific sequences (e.g., legible letter shapes). In adults, the wrist and fingers (distal joints) are mainly responsible for the graphical output [16,17], whereas the shoulder and elbow (proximal joints) are primarily involved in keeping the writing tool on a horizontal plane, and the proximal joints’ angular motion is roughly proportional to the size of the script or drawing being produced [18].

More specifically, there are two typical movements while handwriting. During the first movement, the index finger and thumb are held opposite each other and both move to and from the hand palm (by flexion/extension of all finger joints), which results in back-and-forth movements of the pen tip [16,19]. The second typical movement is the rotation of the entire hand around the wrist through palmar flexion/extension and ulnar abduction as well as movements of the thumb (radial abduction/dorsal flexion) [19]. The four upper limb muscles that are mainly involved in these two movements are thenar (thumb), wrist flexor, wrist extensor and trapezius (shoulder) [20,21]. These two movements are considered the main axes in handwriting, and their effect on the writing process and product has been extensively studied [16,19,22,23]. 

Previous studies have demonstrated that the variability and duration of strokes depend on the movements along these main axes [19]. However, handwriting characteristics, such as pen pressure and the direction of the most frequent handwriting movements, do not correlate with the movements along these main axes [22]. In this context, the proximal–distal principle, which postulates the proximal stability at the shoulder as the prerequisite for the hand use, is crucial [21]. The development of the proximal–distal principle was recently investigated by [24]. They tested children from first to fifth grade by recording their handwriting movements as they drew a loop pattern on a tablet screen. Younger children, especially those in third grade and younger, lifted their forearm and elbow (proximal joints) when continuing the loop pattern, whereas children from fourth grade onwards rather used the rotation of the wrist to execute the same movement. Seemingly, the development of the hand muscles directly depends on proximal motor development.

However, both systems could also function independently of each other. This has been investigated by [21]. They recorded the muscle activity in children during handwriting tasks by using electromyography (EMG) signals to evaluate the muscle activity’s relationship to the quality of the writing product. The authors were specifically interested in the muscle activity of the proximal and distal muscles and their relationship during handwriting tasks. Thirty-five children in the third and fourth grades (mean age 9.7 years) performed a handwriting test (copying words) while their muscle activity at the upper trapezius and thumb (thenar) was recorded. Additionally, the writing speed (number of letters in 1 min) and the legibility (scored on a scale of 1 to 4) was measured. The authors calculated the amount and the variability of muscle activity (coefficient of variation [CV]). Distal muscles indicated a greater amount of muscle activity than proximal muscles. The variability of muscle activity revealed a significantly higher CV in the distal muscles compared with the proximal muscles. There was also a significant correlation between the CV in the distal and proximal muscles, meaning that a low variability of muscle activity in the distal muscles was also reflected in a low variability in the proximal muscles. Regarding the relationship between writing speed and variability in muscle activity, the results revealed weak significant correlations. A low variability in the distal muscles was associated with faster writing velocity (more letters per minute). The authors found no significant results for an association of legibility and muscle activity. They concluded that the proximal muscles function as stabilizers for the distal muscles during handwriting tasks [21]. These authors used only one method to evaluate handwriting in children—namely sEMG—and they evaluated the muscle activity in relation to the handwriting product. We will propose a novel approach and combine sEMG with handwriting process measures to gain more insights into the proximal–distal principle and its involvement in the writing process.

Surface electromyography (sEMG) allows a real-time, noninvasive assessment of the activation pattern of muscles during task performance. More specifically, the assessment of muscle activity through EMG signals provides valuable insights into the graphomotor processes of the upper limb muscles. The assessment of muscle activity during handwriting tasks through sEMG signals has been studied in the past in clinical studies in relation to writer’s cramp [25,26,27]. Writer’s cramp defines the increased inappropriate muscular co-activation of flexor and extensor muscles in the arm during writing [25] or painful muscle cramps in the thumb and two adjoining fingers caused by excessive pressure on the pen [26]. It can lead to individuals experiencing a tighter grip on the pen and to their hand or arm developing an abnormal posture due to discomfort. The authors in [27] successfully treated writer’s cramp in five adult patients with biofeedback-based sensorimotor training using an EMG feedback system.

Researchers and educators are still trying to investigate in more detail the distinction in handwriting movements between beginning, intermediate and advanced writers. The underlying motor programs in the hand-related muscles are still not researched well enough to provide specific handwriting instructions for teachers. The development of specific muscular programs in children that eventually help them to become efficient and fluent writers is of particular interest these days. Existing research methods provide information about the writing process (e.g., on tablets) used in handwriting research, as well as insights into muscle activity during the writing tasks (e.g., with electromyography) mainly applied in clinical research. However, to our knowledge, the combination of both approaches has not yet been proposed. This interdisciplinary application of these two methods and the benefits of the combination of both methods might provide more fine-grained information about the handwriting process and the development of skilled writing. Further, it might help teachers to support handwriting acquisition in educational contexts.

The aim of this study is to fill this research gap and suggest an approach that investigates the muscle activity in proximal and distal joints during handwriting while simultaneously measuring the writing process on a tablet. We measured the muscle activity in four handwriting-related muscles—thumb, wrist extensor, wrist flexor and shoulder—to analyze the differences in muscle activity of the abovementioned muscles of still-developing and fully developed writers. Through this, we explored the link between the handwriting movements and the actual muscles that are involved in producing these movements. By combining both assessments, we strive to obtain more insights into the proximal–distal relationship and how writers at various levels of handwriting skill (children and adults) motorically control their handwriting movements.

Specifically, we studied two groups with different levels of handwriting skill—children in the third and fourth grades in primary school and adults. We decided to focus on children in this age group for several reasons: First, we wanted to investigate intermediate writers and not novices since younger children (second grade or younger) might have had difficulties in executing the writing tasks with proper attention and quality. Second, the preparation of the skin for sEMG recording is quite unusual and appeared unsuitable for younger children. Third, for sEMG recording we needed the children to remain relatively still during the task. Finally, the study by [24] revealed developmental differences in the proximal–distal principle for primary school students. Before third grade, these students were still lifting their forearm and elbow to continue a loop pattern. 

## 2. Materials and Methods

### 2.1. Participants

Thirty-seven children in the third and fourth grades of primary school (25 female, mean age 9.6 years, SD: 0.5) and 18 adults (11 female, mean age 28.6 years, SD: 5.5) participated in this study. The children were recruited from schools in Brandenburg, Germany and adults were students and employees at the University of Potsdam. Seven of the children and three of the adults were left-handed. All participants were naïve to the purpose of the study. They had normal or corrected-to-normal vision. The children’s parents were informed about the study in an information letter and gave written informed consent for the participation of their children. The adult participants were also given an information sheet about the study and gave written and informed consent.

### 2.2. Instruments—Recording Muscle Activity Data

**Figure 1:**
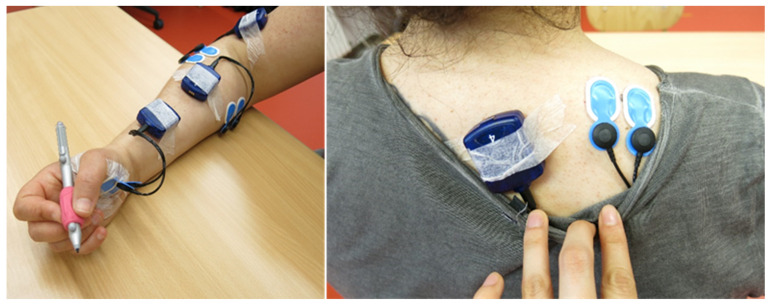
General preparation: Electrode placement on arm (**left** picture) and upper shoulder (**right** picture).

To record the muscle activity of the writing arm and hand, we used a Myon m320RX surface electromyography (sEMG) system for the noninvasive assessment of the neuromuscular system (Myon AG, Kloten, Switzerland). We used four EMG channels to record the signals of four electrodes that were placed in parallel bipolar orientation to the fibers on four muscles of the upper limb. The self-adhesive disposable bipolar silver/silver chloride (Ag/AgCl) electrodes (size 30 × 22 mm, length × width) are specifically developed for children (Ambu^®^ BlueSensor N, Ballerup, Denmark). We placed one electrode on the thumb (thenar: adductor pollicis, abductor pollicis brevis and flexor pollicis brevis), two electrodes on the forearm (the wrist extensor group: extensor carpi ulnaris and the wrist flexor group: flexor carpi ulnaris, palmaris longus) and one electrode on the shoulder (descending part of the trapezius). Figure 1 shows the position of the four electrodes and their Bluetooth boxes. We recorded the EMG signal with a sampling rate of 4000 Hz. It was immediately amplified (gain 1000, bandwidth 5–500 Hz). 

#### 2.2.1. Correct Writing Time Measurement

To assess the correct writing time, it was necessary to include a 3D motion-capturing system (Vicon Vantage 5, Oxford, UK), which tracked the movement of the stylus. We attached three round, grey, reflective markers onto the pen (top, middle and close to the stylus tip, see Figure 1 left picture) to identify the exact time when the stylus touched the tablet’s surface. Eight infrared cameras tracked the movement of the stylus at a frequency of 200 Hz. We processed the motion capturing data synchronously with the EMG signal using Vicon Nexus (version 2.5, Vicon Motion Systems Ltd., Oxford, UK).

#### 2.2.2. sEMG Preparation

**Figure 2:**
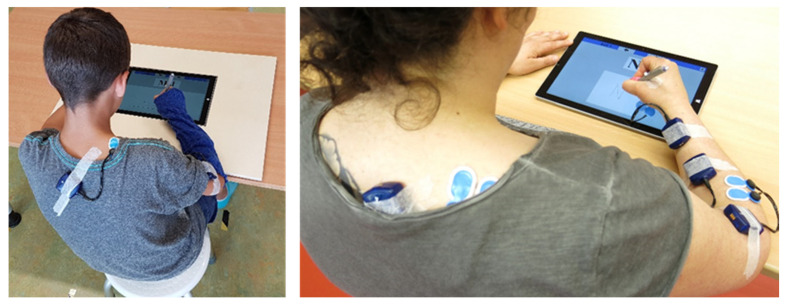
Writing on the tablet with electrodes placed on arm and shoulder (**left** picture: child; **right** picture: adult).

Before placing the electrodes on the arm and shoulder, the children’s skin was cleaned and gently rubbed with a peeling gel, whereas the adults’ skin was shaved and gently rubbed/abrased with sandpaper to obtain an inter-electrode impedance of close to or less than 5 kΩ. Then the skin regions were cleaned with an alcohol solution [28]. The pre-gelled electrodes were placed in parallel orientation to the muscle fiber with a center-to-center spacing of 20 mm. The lightweight EMG amplification and wireless transmitter boxes were attached to the skin with adhesive and skin-friendly tape (see Figure 2). For children, a fabric cuff on the arm was used to cover and secure the EMG setup (see Figure 2, left picture)—this way they were less distracted by the electrodes and Bluetooth boxes. After application of the EMG electrodes, the EMG signal was checked in the resting state and during activity (muscle function tests) to make sure that recording and Bluetooth transmission worked properly.

### 2.3. Instruments—Recording Handwriting Process Data

The x- and y-coordinates of the stylus and the timestamp were recorded during writing. Then, the velocity profiles were smoothed with R scripts, implementing the non-parametrical kernel estimation devised by [29]. 

The following handwriting measures were extracted:

- *Writing velocity* in millimeters per second (mm/s): the first derivative of the x- and y-coordinates with respect to time was taken to obtain the writing velocity [15].
- *Number of inversions in velocity (NIVs)*, which are related to the number of accelerations and decelerations during writing, indicate the level of handwriting automaticity [9]. Low NIVs represent a smooth and automatized handwriting movement, whereas high NIVs suggest less automaticity, for instance during the acquisition of handwriting when movements need to be more controlled [9].
  The mean value for NIVs of each item and participant was divided by the number of item strokes to allow for comparison between tasks (e.g., letters and words). A stroke is a part of the writing product that contains one or more velocity maxima (e.g., P contains two strokes, H contains three strokes).
- *Writing duration* in milliseconds (ms), i.e., the length of time that the stylus touched the tablet surface (pressure above 0). The value of writing duration was normalized by dividing the mean value of each item by the number of item strokes to allow for a comparison between tasks.
- *Pen pressure* of the stylus tip on the tablet surface was used. The tablet (Microsoft Surface 2) recorded the pressure in 1024 levels of sensitivity.

### 2.4. Materials

We developed a copying task with three conditions to measure the muscle activity of simple and familiar writing movements. These conditions are usually used in handwriting research to assess distinct levels of handwriting skill.

1. As *geometric forms*, we chose circle, square, triangle, vertical line, slash and backslash because before children learn how to write they use these forms to experiment with handwriting [30]. Note that these forms are independent of language (in contrast to letters and words).
2. The second condition consisted of copying six *capitalized letters* (C, M, N, S, U and V). Letter copying requires the integration of visual and motor abilities to produce the correct order and direction of strokes [31], and children have practiced writing capital letters since the beginning of their schooling.
3. In the third condition, participants were asked to copy *words* in cursive handwriting (ob, und, Mal, Erde, gelb, Mulde; English: if, and, time, earth, yellow, hollow). These words can be written in one go in cursive handwriting without lifting the pen, a skill the children acquired during their first years of schooling. The stimulus words differed in length since this has been shown to influence the variability of hand and upper limb motion [18].

The three conditions differed in complexity, with increasing demand on writing and muscle activity. We specifically used alphabetic script and German words because these were familiar to our participants.

### 2.5. Procedure

#### 2.5.1. Muscle Function Tests

**Figure 3:**
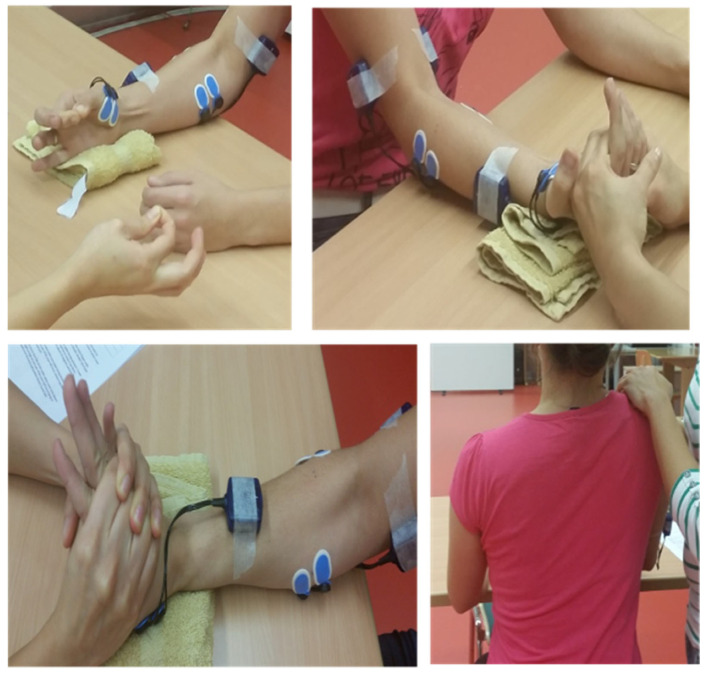
Tasks for maximal/submaximal voluntary contraction to normalize sEMG data for each of the four muscles.

The maximum force in each muscle is individual for each person and muscle; therefore, it is necessary to administer a test of maximal or submaximal voluntary contraction (MVC) for each of the four muscles to normalize the EMG data (see Figure 3). This allows for comparison of muscle activity levels between muscles, tasks and participants [32]. As a reference for normalization, participants needed to perform maximal VC for thumb, wrist extensor and flexor and submaximal unilateral VC for the shoulder [32,33,34]. Only submaximal VC was used for the shoulder to avoid discomfort and possible injuries [33]. Next, we will describe in detail how to perform the voluntary contraction procedure for the four muscles.

For this procedure, participants sat opposite the experimenter at a table with a folded soft towel to increase participants’ comfort of the hand during the procedure. Each of the four MVC tasks lasted for 5 s and was repeated five times with a 20–30 s rest between trials to avoid fatigue. Figure 3 shows the four MVC tasks. For *MVC of the thumb* (upper left picture of Figure 3), participants pressed together the tip of their thumb and index finger with full force. To measure *MVC of the wrist flexors* (upper right picture of Figure 3), participants placed their wrist over a towel with their palm facing upward. They lifted their palm as high as possible. Then, the experimenter put their hands against the participant’s upper palm and participants were asked to pull with full force while keeping their fingers relaxed. For the *MVC of the wrist extensors* (lower left picture of Figure 3), participants placed their wrist over the towel with their palm facing downward. Again, they lifted their palm as high as possible. The experimenter put their hands against the back of the participant’s upper palm. Then, the participants pulled their palm with full force while keeping their fingers relaxed. To assess *submaximal VC for the shoulder* (thenar, lower right picture of Figure 3), the participant sat on a stool, and the experimenter stood on the side of the respective shoulder (writing hand). The experimenter put their hands on the shoulder to build a counterforce. Then the participant was asked to bring their shoulder to meet their ear with half of their full force (shoulder elevation). For each muscle, the three trials with the highest average force were selected and their average value was calculated. This value was used as 100% muscle activity to normalize the EMG data for each participant and muscle (expressed as %MVC).

We used the software proEMG (version 2.1, prophysics AG, Kloten, Switzerland) to manually code when the stylus touched the tablet (start of writing) and when it was lifted (end of writing). We used the motion capture data to pinpoint the exact timing and cut the item appropriately. Next, the EMG signal was filtered using a 2nd-order Butterworth filter (10–400 Hz). The root mean square (RMS) (20 ms moving average) of the raw data in volts was calculated with proEMG. Then, the RMS was normalized to the percentage of muscle activity of each trial (in %MVC) relative to the highest individual muscle activity [32,33].

#### 2.5.2. Data Collection

During data collection, participants sat on a stool adjusted to their height in front of a table on which the tablet was positioned so that their feet touched the ground and their knees were in a roughly 90 degree angle (Figure 2). The experimenter placed the stylus in the middle of the tablet to prevent handedness bias. The participants were instructed to sit in a normal writing position (e.g., upper body upright) while putting their wrist on the tablet and their elbow comfortably on the table (or, for the children, on the frame around the tablet). They were informed that the hand or fingers would not leave a mark on the tablet screen; only the tip of the stylus would trigger a line on the tablet. The experimenter remained in the same room, observed each participant’s task performance and used a protocol sheet to write down any exceptional actions such as strong movements.

One session of data collection took approximately 50 to 60 min in total. The children were tested individually in a quiet room in their school, and the adults were invited to a laboratory at the University of Potsdam. When participants entered the room or laboratory, they were informed about the procedure orally (children) or via an information sheet (adults). Before data collection, children were familiarized with the tablet by writing their first name on a line. Usually, children had never written with a pen on a tablet surface before. By letting them write a familiar word (such as their name) they became acquainted with the way the digital pen moved on the tablet’s surface and how the visual feedback of the writing was displayed.

**Figure 4:**
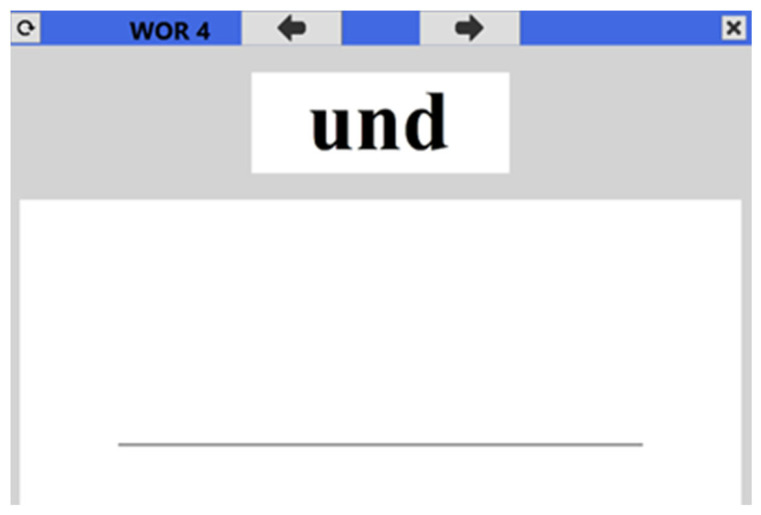
Screenshot of item (item 4 “und”) and response line (for children).

Participants then started with copying the geometric items, followed by copying capitalized letters and writing words. The setup was such that they had to randomly repeat the six items per condition five times. Each condition started with one practice trial. The items were shown in the upper half of the screen and had to be copied into a square directly below (see Figure 4). The experimenter verbally announced the start of each trial, then the participant copied the item. The participant sat upright again while resting their writing hand on the table to show completion of the trial. The experimenter asked the participant to proceed to the next trial by touching the arrow to the right on the upper screen. This procedure was repeated for each trial. 

Each square for the geometric forms and letters had a size of 8.7 × 9.5 cm. The square for the words had the following dimensions: 10.5 × 24.7 cm. For the children, we added a line in both the letter and word condition since they are more used to writing on a line than on an empty space. Figure 4 shows the item *und* (English: and). The acquisition software was programmed in C# and XAML using Visual Studio Community 2013 Update 4 and the Windows Presentation Foundation runtime libraries provided by the Microsoft .NET Framework 4.5© Microsoft.

### 2.6. Data Preprocessing

Each produced item consisted of a csv file (containing the x- and y-coordinates of the pen-tip, the timestamp and pen pressure), a jpg file (the image of the writing product including the background as in Figure 4) and an xlsx file (showing the filtered and RMS-normalized sEMG data for each trial). In a first step, all trials were checked for correct recording of tablet and sEMG data (no missing data points in the csv and xlsx files). If one of the files contained missing data, the complete item was removed from further analyses. In a second step, the jpg file of each trial was visually checked for correct spelling and writing inside of the predefined space. In a third step, the csv files of the tablet data were checked for pen lifts (pressure below 0). In case participants did not follow the instructions correctly (write inside of the white space and do not lift the pen when copying the item), these data were excluded as well. 

The thorough inspection resulted in exclusion of data as follows:

1. Correct spelling of the word: 0.45% misspelled words (adults: 7 items, children: 22 items). Spelling errors were *d* instead of *b*, adding another letter, omitting a letter, starting with a capital instead of a lowercase letter, substituting a letter (*gebb* instead of *gelb*).
2. Correct writing space: 0.49% of data were excluded as they had been drawn or written beyond the predefined writing space (only children: 24 items).
3. Writing in one go: Although participants were instructed to draw or write the items in one go, they sometimes lifted the pen. A total of 2.23% of the data were excluded because the words were written with more than one line, i.e., pen lifts in between the item (adults: 16 items, children: 92 items).

Further data exclusion: Another 0.7% of the data had to be excluded due to technical problems with the sEMG recording (adults: 4 items, children: 34 items). 

After data cleaning, we calculated the mean and standard deviation for each item and detected 0.13% of the data (adults: 5 items, children: 1 item) that had a writing duration of less than 200 ms, which is implausible for a handwriting task. Hence, we excluded these data points. In total, we excluded 4.1% of the data for both groups.

### 2.7. Statistical Analysis 

The participants copied each item five times in a random order. For the statistical analyses, we averaged these five trials to obtain a mean and standard deviation for each item. Then, the data were analyzed in two steps. In a first step, the data for both methods (EMG and handwriting process data) were analyzed separately. We analyzed all data using linear mixed effect models with the lme-function provided by the software R version 4.0.3 [35] and the nlme-package [36]. One model was run for each handwriting measure (log-transformed velocity, NIVs normalized by stroke, writing duration normalized by stroke and pen pressure) and for the muscle activity of each muscle (thumb, flexor, extensor and shoulder). The handwriting measure as well as the muscle activity were used as the dependent variables of the model. Writing velocity was log-transformed to avoid skewed distributions. As independent variables for each model, we used the group variable (adults vs. children). Participants and items were used as random factors in each model (random by-subject and by-item intercepts). For all independent variables, planned contrasts were used to compare the variable levels (e.g., for group as the independent variable, adults and children are compared). To take individual differences into account the data for participants were aggregated. 

In a second step, the EMG and tablet data were combined into one analysis. With this analysis we wanted to figure out if and when both data sets measure the same underlying processes in handwriting. At first, the data were subdivided into muscle type (thumb, wrist extensor, wrist flexor and shoulder). Before running correlations, the Shapiro–Wilk test was performed on each of the four parameters (velocity, normalized NIVs, pressure and muscle activity) to test for a normal distribution. The data in the study were not normally distributed; therefore, we ran Spearman rank correlations. Then, correlation matrices were computed for the average values for participants in terms of velocity, normalized NIVs, pressure and the average of muscle activity to get an overview of the data. These correlational analyses provided subsets of relevant variables as input for further analyses (i.e., linear regression models).

To investigate the contribution of each handwriting-related muscle separately, we used the normalized muscle activity of the four muscles in separate linear mixed effect models as dependent variables. The focus of our analysis was the comparison of the groups to ascertain distinct levels in handwriting skill. We did not differentiate between task demands in the statistical analysis. To combine the data of the two methods—sEMG of muscle activity and tablet data during handwriting tasks—we ran Spearman rank correlations. At first, we subdivided the data for adults and children, then we clustered the items for each participant such that these items represented one data point in the correlational analysis.

## 3. Results

In the following sections we present the results of the data analyses of our study. We start with the results of the muscle activity data recorded with the sEMG (see Section 3.1). In Section 3.2, we will present the results of the handwriting process data: we selected writing velocity, writing duration, number of inversions in velocity and pen pressure as meaningful parameters to describe the level of handwriting skill in our groups of intermediate and advanced writers. In the last paragraphs, we will combine both analyses and present correlational matrices of the sEMG and tablet data. To investigate the relationship between both data sets in more detail, we conducted regression analyses on selected parameters.

### 3.1. Muscle Activity during Handwriting Tasks—sEMG

**Figure 5:**
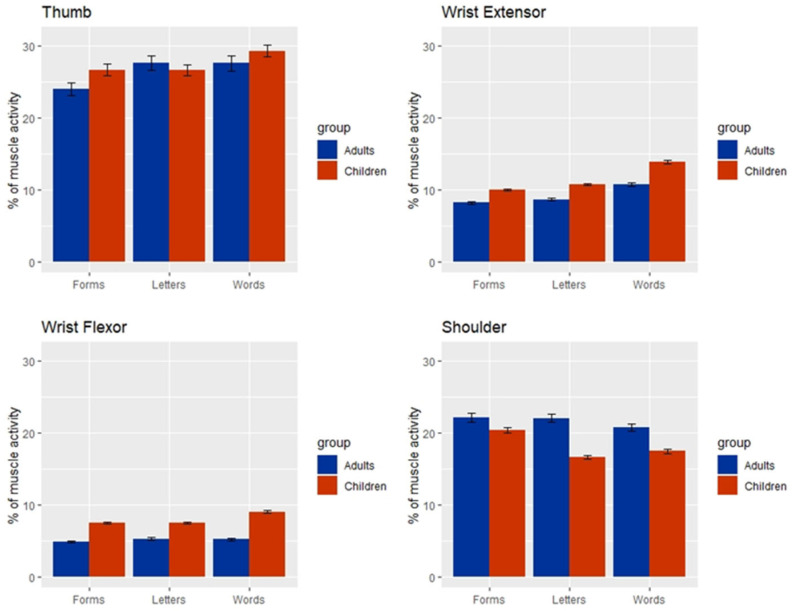
Mean percentage of muscle activity during handwriting tasks for each muscle, task and group (with 95% confidence intervals).

Figure 5 shows the descriptive data for the normalized muscle activity during the three handwriting tasks for both groups and the four muscle groups. Note that the muscle activity had been normalized to the maximal voluntary contraction data and represents the percentage of activity for each muscle separately (%MVC).

We found a higher muscle activity for children compared with adults for the thumb (*t*(2872) = 7.69, *p* < 0.001), wrist flexor (*t*(2875) = 29.16, *p* < 0.001) and wrist extensor (*t*(2875) = 16.15, *p* < 0.001). Only for the shoulder did children reveal a lower muscle activity than adults (*t*(2871) = −16.07, *p* < 0.001). For the children in our study, the proximal muscle activity is lower than for adults, suggesting that they had not yet fully developed the support possibility that the shoulder could offer them during handwriting. Developing writers display a greater variability in muscle activity and show more undesirable movements that might reflect a less developed or suboptimal motor system. Apparently, they need to consciously control their hand movements and require more muscle activity in distal muscles.

### 3.2. Handwriting Process Data 

Table 1 presents the descriptive data for the handwriting process measures of the three tasks and both groups.

The writing velocity was higher for adults than for children (*t*(2875) = −29.34, *p* < 0.001). For the measure of automaticity in handwriting movements, adults generally produced fewer NIVs than children (*t*(2875) = 16.56, *p* < 0.001). The writing duration was always shorter for adults than for children (*t*(2875) = 41.50, *p* < 0.001). Regarding pen pressure, the groups did not differ significantly from each other (*t*(2875) = 1.70, *p* = 0.09).

### 3.3. Correlations between Muscle Activity (sEMG) and Handwriting Process Data

Our combined analysis of the muscle activity (sEMG) and the handwriting process data (tablet) yielded the correlational matrix in Table 2. We chose three handwriting measures (writing velocity, NIVs and pressure) and correlated these with the sEMG data of four muscles (thumb, wrist extensor, wrist flexor and shoulder) in each group.

For adults, we found a significant correlation between pen pressure and the muscle activity in the wrist extensor as well as the wrist flexor. For children, we obtained a significant relationship for the writing velocity and normalized NIVs with the muscle activity in the shoulder. None of the other correlations yielded significant results.

**Figure 6:**
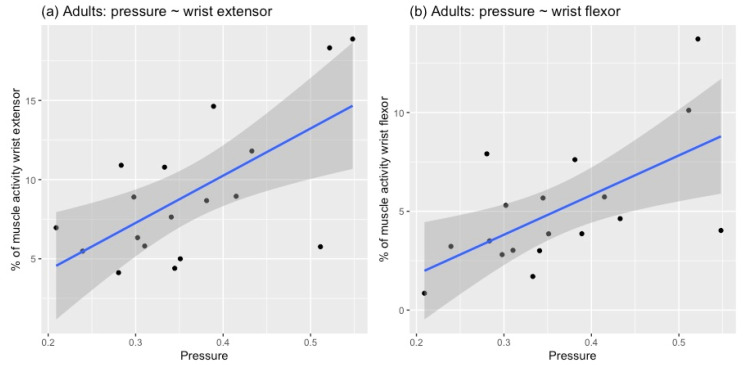
Regression lines with confidence intervals for linear regression models of pen pressure and muscle activity in the wrist extensor (**a**) as well as pen pressure and muscle activity in the wrist flexor (**b**) for the adults.

**Figure 7:**
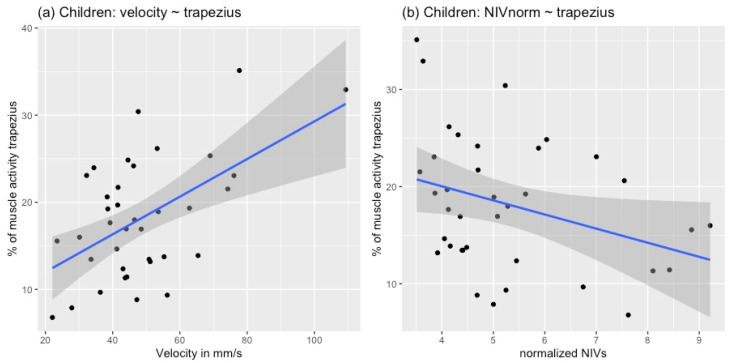
Regression lines with confidence intervals for linear regression models of writing velocity and muscle activity in the trapezius (**a**) as well as NIVs and muscle activity in the trapezius (**b**) for the children.

To investigate these significant relationships in more detail, we conducted linear regression models with these parameters as variables for each group (see Figure 6 and Figure 7). For adults (Figure 6), we confirmed the positive relationship between pen pressure and the wrist extensor muscle activity (β = 0.02, SE = 0.01, *t*(16) = 3.07, *p* = 0.007, R^2^ = 0.4068), as well as the wrist flexor muscle activity (β = 0.01, SE = 0.00, *t*(16) = 3.31, *p* = 0.004, R^2^ = 0.37). The higher the adults’ muscle activity in the wrist flexor or wrist extensor, the higher the pressure of the pen onto the writing surface.

For children (Figure 7), the relationship between velocity and the shoulder muscle activity was confirmed (β = 1.34, SE = 0.35, *t*(35) = 3.77, *p* < 0.001, R^2^ = 0.2889). Further, we found an inverse relationship between normalized NIVs and the shoulder muscle activity (β = −0.08, SE = 0.04, *t*(35) = −2.09, *p* = 0.04, R^2^ = 0.1109). These results demonstrate that the higher the muscle activity in the children’s shoulder, the faster the velocity and the more automated the handwriting movement (lower NIVs).

## 4. Discussion

We measured the muscle activity in four handwriting-related muscles—thumb, wrist extensor, wrist flexor and shoulder—to analyze the differences in muscle activity of the abovementioned muscles in two groups with varying handwriting skills (adults and children). We combined the simultaneous recording of muscle activity of distal and proximal muscles during handwriting tasks using sEMG and handwriting process measures on a tablet.

### 4.1. Differences between Groups in Muscle Activity during Handwriting

When comparing the muscle activity of adults and children, we found greater activity in the children’s distal muscles of the thumb, wrist flexor and wrist extensor and lower muscle activity in the children’s shoulders (proximal) compared with adults (similar to [21]). It seems that in children, distal muscles tend to be used for dynamic movements and proximal muscles function as tonic stabilizers. In our study, the proximal muscle activity was lower for children than for adults, suggesting that they had not yet fully developed the adult-like support possibility that the shoulder could offer during handwriting tasks. In general, the participating children displayed a greater variability in their muscle activity and exhibited more undesirable movements that might reflect a less developed or less efficiently used motor system. Apparently, they needed to consciously control their hand movements and required more muscle activity for task execution in distal muscles compared with adults.

### 4.2. Differences between Groups in Handwriting Process Measures

We replicated previous findings in handwriting research with our method: experienced writers showed a greater writing velocity and lower NIVs than less-experienced writers [10,11]. Interestingly, we did not find a difference in pen pressure for adults and children. Previous research suggests that less automatized writers compensate their handwriting movements with a greater pressure on the writing surface. We did not find this effect in our data. One possible explanation for the missing effect could be the difference in the writing experience of the groups in these studies. In the present study, we compared third and fourth graders with experienced adults. In contrast, Ref. [11] tested preschoolers as well as second graders and [10] researched handwriting in second and ninth graders. All of these child groups have a different level of writing experience and development. Another reason for there being no difference in pressure in our study could be the diverging task demands. Our participants had to perform a set of very different copying tasks such as copying geometric forms, capitalized letters and words in cursive in our study. Previous studies used other tasks (e.g., writing the alphabet, writing after dictation). It is possible that the difference in task demands introduced another variable besides the difference in writing experience. Future studies might take a closer look at these potential influences on handwriting movements.

### 4.3. Correlations between Groups in Muscle Activity and the Writing Process Measures 

When linking the muscle activity data obtained from the sEMG with handwriting process measures recorded by the tablet in a correlational analysis, we see a developmental pattern. The groups differed only in correlations regarding the wrist extensor and flexor (distal) and the shoulder (proximal) muscles. Adults exhibited a relationship between pen pressure and the muscle activity in the wrist extensor and wrist flexor. The higher the pressure of the pen during writing, the higher the muscle activity in both areas. The analysis of the data from the children revealed a negative correlation between the shoulder and handwriting automaticity as well as a positive relationship between the shoulders’ muscle activity and writing velocity. The more automatized (lower NIVs) and the faster the children wrote, the higher the muscle activity in the shoulder.

A more economical distal muscle activity (lower variability) in adults was associated with a faster writing velocity (mm per second). Similar results were obtained by [21], who found the same relationship using letters per minute as the measure for writing speed. The faster the adult participants were when writing letters per minute, the lower their variability in muscle activity in the distal muscles. This relationship can be explained by the fact that the motor system attempts to perform a task, such as handwriting, in the most efficient and economical manner with the available resources, which translates to a low muscle activity in experienced writers. Handwriting becomes more efficient and automatic and less muscle activity and variability is perceptible only when all muscle types are balanced out, such as in adults. 

Our combined method replicates these results for writing process measures. We found significant relationships between the wrist extensor and wrist flexor and the pressure of the writing tool onto the tablet for adults. It seems that the adults used the distal muscles to control the writing output itself [16,17], whereas their shoulder seemed to be less involved in writing. The intermediate writers in our sample study (children) controlled their handwriting movements predominantly with their proximal muscles (shoulder), which yielded significant relationships with writing velocity and automaticity (NIVs). This could be an argument for the proximal muscles still serving as a stabilizer for the handwriting movements [21]. It is possible that intermediate writers seem to be focused more on keeping the writing tool on the surface of the tablet and concentrate less on the writing product itself. It seems that during handwriting development the graphomotor control shifts from the shoulder (proximal muscles) toward the lower arm—wrist extensor and flexor. Another explanation could be that adults are more focused on copying the items more accurately, whereas children are more concentrated on keeping the pen steady on the smooth surface of the tablet. 

This research shows that the combination of writing process measures and the recording of muscle activity in hand-related muscles yields a new perspective on underlying graphomotor processes and their development.

## 5. Limitations and Future Directions

The impact of our study is, however, still limited by the fact that we used a small sample, which does not allow us to generalize to a larger population. Further, it needs more research on various age groups (e.g., early writers) to determine the actual development of graphomotor processes.

Another limitation of the study might be the order of the tasks. We chose to order the tasks in relation to their complexity, starting with an easy task of copying geometric shapes, then moving on to writing letters and finally writing words in cursive handwriting so that the participants had to write the word in one go (i.e., the pen touches the surface when starting to write the word and is lifted only after having finished writing that word). The purpose of the ordering of tasks was to familiarize the participants more and more with writing on the tablet surface as the experiment progressed. However, this ordering might have introduced other factors such as fatigue or habituation. Therefore, we refrained from statistically comparing between-task performance. Furthermore, we considered only one proximal muscle (trapezius) since we based our selection on previous sEMG studies that investigated tasks that were similar to handwriting; however, there might be more muscles involved in handwriting movements that are worth investigating. Moreover, we decided to analyze average values for the muscle activity and handwriting process measures. We could also have used the maximum value of the values, the duration of an EMG burst, the coefficient of variation for the parameters or the combination of several muscle groups to investigate coordination patterns of several involved muscles (We thank the reviewers for these suggestions.). This would have given a more detailed picture of the contribution of different muscle groups or developmental aspects of handwriting.

## 6. Conclusions

Our results suggest a clear differential involvement of proximal and distal joints during handwriting tasks on a tablet for varying levels of handwriting automaticity. The combined analysis of the handwriting process—muscle activity and tablet data—allows to directly link the graphomotor execution in the handwriting muscles and the actual performance of the pen on a tablet. Researchers can use this method to disentangle the exact relationship between writing process measures, handwriting quality and muscle activity during handwriting tasks, especially in developing and novice writers. It might also be of use for teachers in schools for efficient instructions on how to involve rather distal muscle groups (in the fingers and hands) instead of using the proximal muscle (such as the shoulder) during writing. An efficient use of handwriting muscles is the prerequisite for fluent and automatized handwriting performance. Further, our method could be used to diagnose children with specific handwriting problems (e.g., writer’s cramp, DCD) to provide a detailed analysis of the involved muscles and detect challenges in handwriting acquisition before they arise.

## Figures and Tables

**Table 1:** Means and standard deviations in parentheses for the handwriting process measures for adults (*n* = 18) and children (*n* = 37).

	Geometric Forms	Letters	Words
**Writing velocity mm/s** Adults Children			
	59.2 (29.3)	85.1 (38.5)	70.4 (24.8)
	47.3 (28.4)	47.4 (26.6)	48.8 (22.0)
**NIVs (divided by no. of strokes per item)** Adults Children			
	4.3 (2.4)	3.8 (2.2)	2.1 (0.3)
	6.6 (4.0)	6.3 (4.7)	2.8 (0.7)
**Writing duration in ms (divided by no. of strokes per item)** Adults Children			
	746.1 (357.9)	550.3 (353.1)	230.9 (68.4)
	1183.9 (605.4)	1124.6 (693.2)	458.1 (148.3)
**Pen pressure** Adults Children			
	0.356 (0.12)	0.359 (0.11)	0.373 (0.10)
	0.369 (0.15)	0.351 (0.15)	0.391 (0.14)

**Table 2:** Correlations between the muscle activity (normalized sEMG, %MVC) and handwriting process data (tablet data) for each group (adults *n* = 18 and children *n* = 37), with confidence intervals in square brackets.

	Thumb	Wrist Extensor	Wrist Flexor	Shoulder
**Adults** Velocity mm/s NIVs (norm.) Pen pressure	0.20 [−0.30, 0.61]	0.32 [−0.18, 0.68]	0.29 [−0.20, 0.67]	0.25 [−0.24, 0.65]
	0.18 [−0.31, 0.60]	−0.02 [−0.48, 0.45]	−0.40 [−0.73, 0.08]	−0.03 [−0.49, 0.44]
	0.44 [−0.04, 0.75]	0.50 * [0.04, 0.78]	0.56 * [0.13, 0.82]	−0.09 [−0.53, 0.40]
**Children** Velocity mm/s NIVs (norm.) Pen pressure	−0.11 [−0.41, 0.23]	0.25 [−0.08, 0.53]	0.02 [−0.31, 0.34]	0.36 * [0.04, 0.61]
	0.03 [−0.29, 0.35]	−0.12 [−0.43, 0.21]	0.08 [−0.25, 0.39]	−0.33 * [−0.59, −0.01]
	0.26 [−0.07, 0.54]	0.10 [−0.23, 0.41]	0.19 [−0.14, 0.48]	−0.10 [−0.41, 0.23]

Note: * indicates significance at *p* < 0.05.

## Data Availability

The data presented in this study are available on request from the corresponding author.
